# invariant Natural Killer T cell therapy as a novel therapeutic approach in hematological malignancies

**DOI:** 10.3389/frtra.2024.1353803

**Published:** 2024-05-06

**Authors:** Chaiyaporn Boonchalermvichian, Hao Yan, Biki Gupta, Anabel Rubin, Jeanette Baker, Robert S. Negrin

**Affiliations:** Division of Blood and Marrow Transplantation and Cellular Therapy, Department of Medicine, Stanford University, Stanford, CA, United States

**Keywords:** invariant natural killer T cell, graft vs. host disease, acute myeloid leukemia, chimeric antigen receptor, microenvironment, immune therapy, hematopoietic stem cell transplant (HSCT)

## Abstract

Invariant Natural Killer T cell therapy is an emerging platform of immunotherapy for cancer treatment. This unique cell population is a promising candidate for cell therapy for cancer treatment because of its inherent cytotoxicity against CD1d positive cancers as well as its ability to induce host CD8 T cell cross priming. Substantial evidence supports that iNKT cells can modulate myelomonocytic populations in the tumor microenvironment to ameliorate immune dysregulation to antagonize tumor progression. iNKT cells can also protect from graft-versus-host disease (GVHD) through several mechanisms, including the expansion of regulatory T cells (Treg). Ultimately, iNKT cell-based therapy can retain antitumor activity while providing protection against GVHD simultaneously. Therefore, these biological properties render iNKT cells as a promising “off-the-shelf” therapy for diverse hematological malignancies and possible solid tumors. Further the introduction of a chimeric antigen recetor (CAR) can further target iNKT cells and enhance function. We foresee that improved vector design and other strategies such as combinatorial treatments with small molecules or immune checkpoint inhibitors could improve CAR iNKT *in vivo* persistence, functionality and leverage anti-tumor activity along with the abatement of iNKT cell dysfunction or exhaustion.

## 1 Introduction

Invariant Natural Killer T cells (iNKT cells) belong to a subset of innate lymphocytes that express an invariant T-cell receptor (TCR) that recognizes specific lipid antigens presented by cells expressing the MHC-like molecule CD1d (1). iNKT cells are known to have cytotoxicity against cancer cells as well as immunoregulatory like properties where unlike T cells, iNKT cells do not cause GVHD (2). Furthermore, there are several lines of evidence supporting the suppression or prevention of GVHD by iNKT cells through the expansion of CD4 + CD25 + FOXP3 + regulatory T cells (Treg) (3). These unique biological properties render iNKT cells as an ideal “off-the-shelf” product for allogeneic adoptive cell therapy for various hematologic malignancies and perhaps other cancers (4). Chimeric antigen receptors (CARs) can be introduced into iNKT cells to augment cytotoxicity (4–8). Further advantages of CAR iNKT cells are that they are distinct cells that can be isolated and expanded, recognize a unique marker CD1d that is frequently expressed on hematological malignancies and have cytotoxic capacity that may be augmented through other ligands (1, 5, 9). Interestingly, these engineered iNKT cells can exert both direct antitumor activity as well as an indirect activation of host CD8 mediated immunity and do not cause untoward effects such as GVHD (10). In this review, we discuss the biology of this unique cell population, their interaction with other immune cells, and the potential strategies to harness their biological properties to abate immunological dysregulation in hematological malignancies and their complications (1) such as GVHD and to treat disease and prevent relapse.

## 2 iNKT—basic biology

iNKT cells are a unique cell population that shares properties of both T cells and natural killer cells (1, 11). They express an invariant TCR (define mouse and human) defined by the specific interaction between their semi-conserved T cell receptors and either self or exogenous lipid antigen presented by CD1d (a conserved polymorphic MHC class I like molecule) on antigen presenting cell such as dendritic cells (1, 12). CD1d restriction defines this cell population (1, 2). In mice, iNKT cells TCR expression includes Vα14Jα18 chain paring with a limited Vβ repertoire (Vβ2, Vβ7, Vβ8.1, Vβ8.2 or Vβ8.3) (13, 14). In humans, Vα24Jα18 chain pairs almost exclusively with Vβ11. Like conventional T cells, iNKT cells develop in the thymus (1, 15). The double positive (CD4+ CD8+) thymocytes act as progenitor cells for all lymphocytes that belong to the αβ T cell lineage (16). Thymic selection shapes the development and differentiation of functional iNKT cells through the expression of promyelocytic leukemia zinc finger protein or PLZF (17). In C57 BL/6 mice, iNKT cells represent about 1%–2% of lymphocytes in the spleen or liver (13, 14). Mature iNKT cells are widely distributed in various tissues such as bone marrow, gastrointestinal tract, liver, and adipose tissue (13, 18). However, iNKT cells rarely recirculate as compared to MHC restricted T cells (1). In humans, iNKT cells consist of 0.1%–0.2% of T cells and are enriched in omentum (19–22). There are two fundamental lipid antigens that stimulate iNKT cells (1)—(1) glycosphingolipids or ceremide-based glycolipids and (2) glycerol-based lipids such as membrane phospholipids as self-antigens (1). The glycosphingolipid has α-orientation of glycosidic linkage between the carbohydrate head group and the lipid backbone, and this is not known to exist in mammals (1). However, this configuration is found in various pathogens (1, 23–26). This may explain how iNKT cells are stimulated during infection (1, 26). The lipid antigens exert their interaction with CD1d and TCR by regulating the strength of binding of this complex rather than the specificity of interaction (1). The first well characterized lipid antigen is α-galactosylceramide (α-GalCer) which is derived from a murine sponge (27). This is a well-known compound used to stimulate and enhance the expansion of iNKT cells *in vitro* (5). Unfortunately, a-GalCer has shown limited therapeutic efficacy in several clinical trials of cancer treatment but may be an effective strategy to reduce or ameliorate acute GVHD (28–30). Subsequently, there is a newly synthesized non-glycosidic analog—Threitolceremide-6 which potentially enhances anti-tumor activity and renders a potent stimulation of iNKT cells (31). This compound has now been investigated in clinical trials as a potential strategy to enhance the antitumor activity of iNKT cells (32, 33).

CD1d, it is a transmembrane protein like MHC class I that binds non-covalently to β-microglobulin (1). CD1d expression is found mainly on immune cells such as dendritic cells, macrophages, granulocytes, and B cells (34–36). CD1d is also frequently and strongly expressed on various hematological malignancies such as acute myeloid leukemia (AML) (37–39). This hypothesizes that iNKT cells may be an excellent candidate for adoptive cell therapy for hematological malignancies since all individuals have identical CD1d molecules (2). Further, iNKT cells are amendable to the adoptive cell transfer across MHC barriers without significant alloreactive consequences (2). This cell population has a unique immunoregulatory role that redirects immune response to both internal and exogenous signals (1). Several lines of evidence support the concept that iNKT cells can elicit hybrid immune responses encompassing both innate and adaptive immunity (1). iNKT cells have direct cytotoxicity against cancer (1, 2, 4, 40). Furthermore, the transcriptional profiles of iNKT cells are comparable to those of both innate and adaptive immune cells depending on the antigens or stimuli (1). Substantial compelling evidence indicates that iNKT cells can prime CD8 T cells (2, 10). iNKT cells can also interact with other immune cells such as dendritic cells, macrophages, and B cells to mediate the complex adaptive-innate immunological interactions in response to various antigens such as pathogens, cancer cells or other antigens (1). In the next section, we describe iNKT cells subsets and their development in details.

## 3 iNKT cell subsets and their development

The heterogeneity of iNKT cells has been extensively investigated in rodents which suggest three primary iNKT sublineages: iNKT1, iNKT2, and iNKT17 (41). iNKT cells differentiate during murine thymic development into these three distinct subsets that are characterized by their transcriptomic and epigenomic differences (42). iNKT1 cells can be identified by their production of interferon-gamma (IFN-*γ*) and dependence on the cytokine IL-15 for survival (43). iNKT1 cells express the transcription factor T-bet and are characterized by cell surface expression of CXCR3, CCR5, and VLA-1. iNKT2 cells are characterized by their production of interleukin-4 (IL-4) and expression of Gata-3 transcription factor as well as CCR4 and CCR9 surface receptors. iNKT17 cells produce IL-17 and express the ROR-*γ* transcription factor as well as the cell surface markers CCR6, Itgb4, Itgb5, and Itgb7 (44). While the differences in transcription factors maintain cellular identity, the differences in molecular phenotype drive unique tissue distributions (45). Murine iNKT1 cells are generally found in the liver and spleen, iNKT2 cells are most prevalent in the spleen and lymph nodes, and iNKT17 cells are generally located in the lymph nodes and lungs (46). Tissue residence of iNKT cells is long lived, enabling a rapid specific response to local stimuli.

The development of iNKT cell subsets and the signals regulating the commitment of these subsets have been thoroughly reviewed (47–50). Thymic positive selection of iNKT cell precursors depends on recognition of “self” lipid-CD1d complexes by their TCR or presentation of endogenous ligands *α*-galactosylceramide (*α*-GalCer) and *α*-glucosylceramide. iNKT cells are positively selected at the CD4^+^ CD8^+^ double-positive (DP) stage by CD1d-expressing DP thymocytes leading to “stage 0” which is characterized by CD69^+^ CD24^hi^ cells with high Egr2 expression (48, 49). As these cells continue to mature they reach stage 1 characterized by down regulation of CD24 and CD69 and expression of high levels of PLZF (48, 51, 52). These stage 1 iNKT cells can be differentiated based on expression levels of IL-17RB which is selectively expressed on mature iNKT2 and iNKT17 cells but not iNKT1 cells (53). Stage 2 iNKT cells are characterized by acquisition of memory-like CD44^hi^ phenotype, where they differentiate into distinct sub-populations (stage 3): iNKT1 cells, which cease proliferation and acquire T-bet and NK-like characteristics (NK1.1^+^ and CD122^hi^) with downregulation of PLZF and GATA3, iNKT2 cells, which exhibit retention of PLZF and high expression of GATA3 and produce IL4 and IL13 upon stimulation, and iNKT17 cells, which are characterized by upregulated expression of RORgt, intermediary expression of PLZF and downregulated expression of T-bet and produce IL-17 upon stimulation (48). The stages of development of thymic iNKT cells have been summarized in Table 1. Despite exhaustive studies into iNKT cell development and identification of roles of different signaling pathways and transcription factors, the knowledge regarding mechanisms controlling the differentiation of the iNKT subsets remains elusive.

**Table 1:** Development of thymic iNKT cells in mice.

Stage of development	Surface expression	Features
Stage 0	CD69^+^	iNKT cells positively selected at DP stage express high Egr2 levels
	CD24^hi^	
Stage 1	CD69^−^	iNKT cells differentiate into IL-17RB^+^ and IL-17RB^−^ cells that express high levels of PLZF
	CD24^−^	
	CD44^low^	
Stage 2	CD69^−^	IL-17RB^+^ cells retain high PLZF expression and acquire high levels of GATA3 expression (iNKT2) or retain intermediary levels of PLZF and express high levels of RORgT (iNKT17)
	CD24^−^	
	CD44^high^	
Stage 3	CD44^high^	IL-17RB^−^ cells downregulate PLZF and GATA3 expression & acquire T-bet expression (iNKT1); iNKT cells exhibit differentiation into 3 distinct subsets.
	NK1.1^+^	
	CD122^high^	
	CD69+	

## 4 iNKT cell activation

The semi-invariant TCR enables iNKT cells to swiftly respond to specific antigens presented by CD1d molecules (54). CD1d-mediated lipid antigen presentation is a critical process in iNKT cell activation (55, 56). While TCR recognition of glycolipids by CD1d remains central to iNKT cell activation, non-TCR signaling, including cytokine-mediated activation and direct glycolipid binding to CD1d, plays an essential role in modulating iNKT cell responses (46, 57). Understanding the multifaceted mechanisms underlying iNKT cell activation is crucial for harnessing the potential of these unique immune cells in therapeutic and immunomodulatory applications.

### 4.1 CD1d mediated lipid antigen presentation

The nature of the lipid antigens presented by CD1 molecules necessitates specific mechanisms for their uptake by antigen-presenting cells (APCs) and loading onto CD1 molecules (58). Lipid transfer proteins, including apolipoprotein E and fatty acid amide hydrolase could facilitate antigen presentation by CD1d (59–61). Similar to MHC antigens, to generate bioactive fragments lipid antigens need be processed in the lysosome (62). CD1d can present a wide range of antigens, including synthetic antigens (62), microbial antigens (63), and self-antigens (59). α-Galactosylceramide (α-GalCer), a synthetic glycolipid derived from galactosylceramides found in the marine sponge Agelas mauritianus, serves as a potent agonist and is widely used to investigate CD1d-mediated iNKT cell activation (64, 65). Studies using CD1d-deficient mice demonstrated the indispensable role of CD1d in presenting α-GalCer to iNKT cells (12, 66). Thus, a-Galcer is a tool to study and optimize CD1d-mediated iNKT activation.

iNKT cells, recognized by their expression of the NK cell receptor NK1.1, exhibit a repertoire of receptors akin to natural killer (NK) cells (67). This characteristic led to their initial classification as “NK T cells.” This nomenclature created confusion concerning the involvement of cytokines like IL-12, IL-18, and IFN-α in iNKT cell stimulation, given the dependence of NK cell activation on these cytokines (68). However, extensive *in vivo* and *in vitro* data indicate that initial iNKT cell activation is cytokine-independent. Tools such as CD1d tetramers have enabled the discrimination between iNKT cells and NKT cells, providing clear evidence that inflammatory cytokines and co-stimulatory molecules are dispensable for iNKT cell activation (69, 70). In vitro studies using soluble CD1d coated on plates demonstrated that agonist glycolipids bound to CD1d were sufficient to activate iNKT cells (71). Microbial glycolipids from α-proteobacteria could also directly activate iNKT cells without TLR or IL12 (23, 24, 26, 72). It is important to note that excessive iNKT cell activation with analogs can lead to iNKT cell anergy (73–75).

### 4.2 Activation of iNKT cells by non -TCR signaling

iNKT cells express various cytokine receptors, such as IL-2R, IL-7R, IL-12R, and IL-18R, and are capable of rapid cytokine production due to the presence of cytokine transcripts (57, 76, 77). iNKT cells can release IFN-γ when stimulated by cytokines like IL-12 and IL-18 without TCR involvement, suggesting a potential NK-like cell role for iNKT cells (78, 79). During certain infections, iNKT cells can become activated, characterized by the upregulation of CD25 and robust IFN-*γ* production (80, 81). The invariant TCR recognition mediated by CD1d plays a crucial role in this process as either anti-CD1d antibodies or iNKT cell adoptive transfer into CD1d−/− hosts could diminish activation (82). Infections like MCMV activate dendritic cells via TLR9, leading to IL-12 production, which subsequently activates iNKT cells to produce IFN-*γ* (81). TLR9 deficiency in mice infected with MCMV does not lead to significant iNKT cell activation, emphasizing the role of TLR9 in this type of activation (81). Similar findings have shown that CpG-induced iNKT cell activation is dependent on IL-12, with CD1d playing a minimal role (83). Additionally, LPS, another TLR ligand, has shown that CD1d is not always essential in all conditions (84). To sum up, iNKT cells can effectively respond to some pathogens with minimal TCR engagement.

IL-33, a member of the interleukin-1 family, has been shown to promote iNKT cell activation in both humans and murine models (85–87). This cytokine promotes IFN-*γ* production by iNKT cells in the presence of CD1d-mediated α-GalCer stimulation. Notably, when IL-12 is introduced into the system, IL-33 can induce IFN-γ production from iNKT cells even without TCR engagement.

## 5 Interaction of iNKT cells with other immune cells

**Figure 1:**
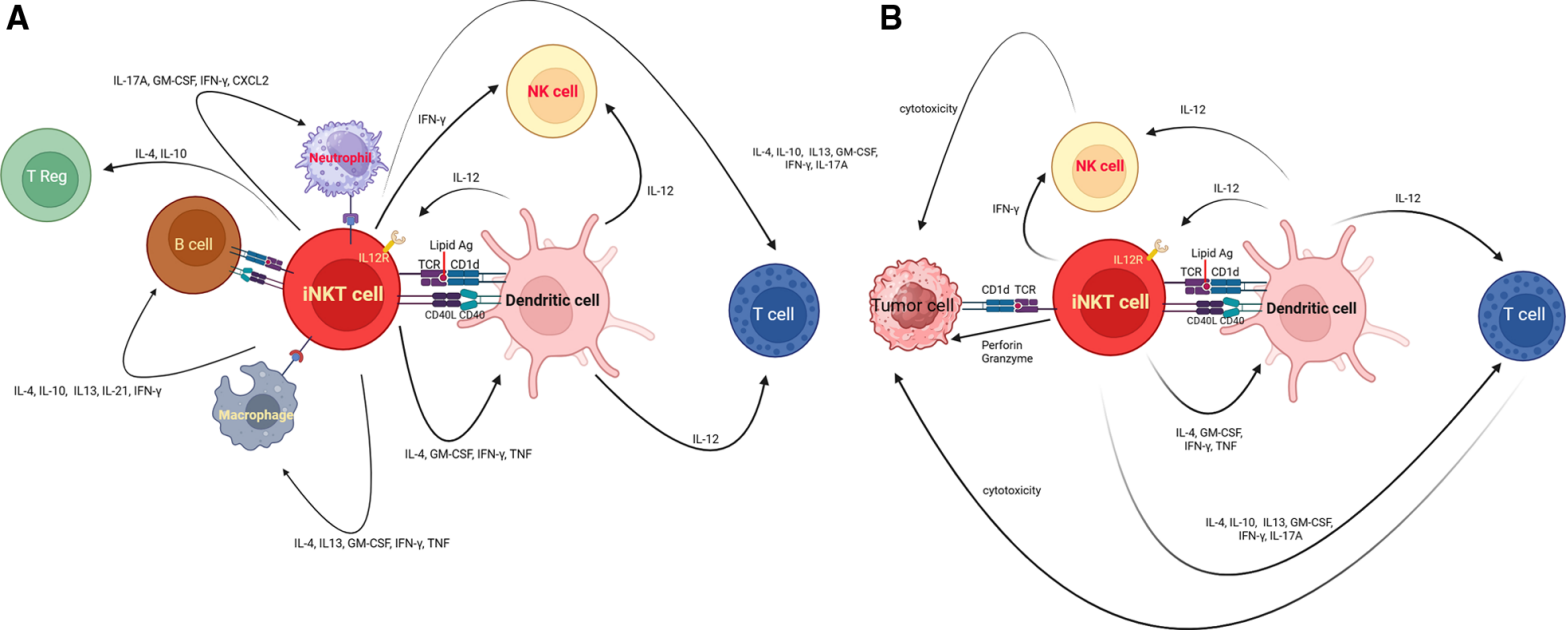
(**A**) Illustrates the interaction between iNKT cells, and other immune cells. iNKT cells possess T-cell receptor (TCR) that recognizes specific lipid antigens presented by cells expressing CD1d. Non-TCR signaling, including cytokine-mediated activation and direct glycolipid binding to CD1d, are sine qua non of modulating iNKT cell responses. In addition, iNKT cells express a variety of receptors, including cytokine receptors such as IL-12R, as well as modulatory molecules like PD-1 and CD40l. iNKT cells interact with other cells such as dendritic cells, their reciprocal interaction results in activation of dendritic cells, triggering IL-12 production and an upregulation in stimulatory lipid antigen presentation for iNKT cells. Upon recognition of self or foreign lipid antigens presented by CD1d on APCs, iNKT cells engage in CD40–CD40 ligand interactions, further enhancing IL-12 production by dendritic cells. The interaction between iNKT cells and other immune cells results in the complex immune response such as NK cell transactivation, T cell activation/differentiation, recruitment/regulation of neutrophils, activation of B cells, regulation of macrophages in tumor microenvironment, or expansion of T regulatory cells. Taken together, iNKT cells act as the mediator of adaptive and innate immune response to either exogenous or self-antigen. (**B**) iNKT cells and immune dysregulation in hematological cancers. iNKT cells eliminate cancer by two main mechanisms. The first mechanism is to directly recognize and eliminate cancer cells harboring CD1d via perforin/ granzyme. The other main mechanism that iNKT cells use to control tumor is indirect crosstalk with other immune cells in the tumor microenvironment (TME). iNKT cells can promote dendritic cell maturation. iNKT cells can also secrete IL-12 which activates anti-tumor CD4+ and CD8+ T cells.

iNKT cells, characterized by their distinctive T Cell Receptor (TCR) composition, possess a remarkable ability to engage with CD1d-expressing antigen-presenting cells (APCs), including dendritic cells (DCs), macrophages, neutrophils, and B cells. This interaction is not only characterized by the recognition of specific antigens but also involves the intricate interplay of cytokines, chemokines, and surface molecules. These multifaceted interactions empower iNKT cells to participate actively in the immune regulatory network (Figure 1A).

### 5.1 Interactions with dendritic cells

Dendritic cells, a vital component of the immune system, constitutively express CD1d, making them pivotal in mediating iNKT cell activation, especially within the spleen (88, 89). Upon presentation of lipid antigens to iNKT cells, an immune response ensues characterized by robust IFNγ production and NK cell transactivation. Notably, a specific subset of dendritic cells, the CD8a + DEC-205+ DCs, stands out for their ability to capture and present diverse glycolipid antigens, including various forms of a-galactosylceramide, leading to distinct cytokine responses (90). Post glycolipid presentation, these DCs dynamically modulate their expression of costimulatory and coinhibitory molecules, a process intricately dependent on antigen structure.

The interaction between these two cell types is bidirectional, particularly during infection. Signals from pattern-recognition receptors activate DCs, triggering IL-12 production and an upregulation in stimulatory lipid antigen presentation for iNKT cells (91, 92). Upon recognition of self or foreign lipid antigens presented by CD1d on APCs, iNKT cells engage in CD40–CD40ligand interactions, further enhancing IL-12 production by DCs. Resting iNKT cells express the IL-12 receptor, with its expression intensifying in response to dendritic cell-derived IL-12 (93, 94). This interaction cascade leads to NK cell transactivation (95), heightened responses to protein antigens by MHC-restricted CD4+ and CD8+ T cells (96), and the licensing of dendritic cell cross-presentation (97).

These bidirectional interactions highlight the cooperative synergy between iNKT cells and dendritic cells, amplifying innate and adaptive immune responses. The ability of activated iNKT cells to facilitate adaptive T cell responses holds significant clinical implications, underscoring the importance of understanding these intricate immunological mechanisms for therapeutic advancements.

### 5.2 Interactions with macrophages

The intricate communication between iNKT cells and macrophages is orchestrated through multiple molecular interactions. The semi-invariant TCR of iNKT cells engages with lipid-loaded CD1d molecules on macrophages (98). This interaction is complemented by co-stimulatory signals mediated through CD40-CD40l and CD80/CD86-CD28 axes (94, 99). Additionally, both cell types secrete a spectrum of pro- and anti-inflammatory cytokines, further enhancing their communication network. In the liver, Kupffer cells, specialized macrophages, are pivotal for iNKT cell activation and clearance of pathogens, exemplified during B. burgdorferi infection, a microorganism producing an α-linked lipid antigen recognized by iNKT cells (100). Lymph node macrophages could also mediate iNKT cell activation. For example, CD169+ subcapsular sinus macrophages present lipids derived from particulate antigens to iNKT cells in the paracortex (101). Thymic macrophages play essential roles in activating and shaping effector functions of thymic iNKT cells (102), demonstrated by the decrease in NKT2 abundance and IL-4 production upon deficiency of thymic F4/80+ Mertk + macrophages or abrogation of CD1d expression in thymic CD11c + cells (103). In the intestine, macrophages derived from murine embryonic and non-bone marrow sources influence the local establishment of iNKT cells in the colon during early life, impacting susceptibility or resistance to iNKT cell-associated mucosal disorders in later stages (104).

On the flip side, activated iNKT cells exert regulatory control over macrophages. In the lean state, iNKT cells aid in dampening adipose inflammation by polarizing macrophages towards an M2 phenotype, a regulatory function impaired in obesity (105, 106). Moreover, iNKT cells modulate tumor growth, partly by altering the phenotype of tumor-associated macrophages, a cell population crucial for the growth of certain neoplasms (107, 108). This bidirectional crosstalk between iNKT cells and macrophages underscores their intricate regulatory roles in immune responses, inflammation, and disease progression.

### 5.3 Interactions with B cells

iNKT cells play a crucial role in regulating B cell functions through cytokine release and CD40 ligand signaling (102, 109). Notably, all B cells express CD1d, with marginal zone B cells (MZBs) in both mice and humans showing particularly high CD1d expression (110). B cells can present foreign or self-lipids on CD1d following B cell receptor-mediated uptake. iNKT cells offer cognate B cell help when their stimulatory lipid antigen is linked to a specific B cell epitope (111). This interaction facilitates the internalization of the antigen complex, enabling the presentation of the stimulatory lipid by CD1d on the same B cell. Additionally, in an inflammatory environment, iNKT cells can provide non-cognate B cell help (112). For instance, co-administration of αGalCer with a protein antigen allows iNKT cells to recognize lipids separately from B cell antigen recognition, ensuring efficient B cell help.

Moreover, iNKT cells contribute to protection against autoimmunity by supporting regulatory B cell (Breg) responses. Activation of iNKT cells by αGalCer expands innate IL-10 producing MZBs, which suppress autoimmunity through IL-10 secretion (113, 114). In a reciprocal manner, Bregs induce iNKT cells to produce IFNγ and modulate cytokine production, reducing Th1 and Th17 responses and consequently ameliorating experimental arthritis (115). The essential role of B cell presentation in iNKT cell regulatory responses is highlighted by studies in mice lacking CD1d on B cells, which exhibited exacerbated arthritis, emphasizing the contribution of activated B cells in engaging the iNKT regulatory pathway. Human studies corroborate these findings, underscoring the clinical relevance of these regulatory interactions (116).

### 5.4 Interactions with neutrophils

In certain infections, iNKT cells recruit neutrophils to infected tissues, a process facilitated by the secretion of chemokines like CXCL2 (117, 118). Moreover, iNKT cells have demonstrated their regulatory function in neutrophil recruitment during events such as ischemia–reperfusion injury and exposure to ozone (119). Additionally, activated iNKT cells exhibit rapid release of IL-4, which enhances neutrophil survival and contributes to hepatitis (120). Paradoxically, these activated iNKT cells also produce IFN-γ in a sequential manner, initiating a negative feedback loop that ameliorates iNKT hepatitis by inducing neutrophil apoptosis. Conversely, heightened concentrations of neutrophils suppress the iNKT cell response in both mice and humans (121). Peripheral Vα14iNKT cells from mice with spontaneous neutrophilia exhibited diminished cytokine production in response to the model iNKT cell antigen αGalCer. Furthermore, these cells displayed lower expression levels of the transcription factors T-bet and GATA3 compared to wild-type controls. Similarly, iNKT cells from the human peritoneal cavity exhibited reduced transcription factor levels during neutrophilic peritonitis, underscoring the intricate interplay between iNKT cells and neutrophils in the immune response (121).

### 5.5 iNKT cells as the mediator of adaptive and innate immune response

The immune system safeguards the organism by detecting and regulating various danger signals, whether originating from within the body or from external sources. This intricate defense mechanism requires a harmonious coordination between the innate and adaptive immune responses, with each equipped to protect the organism in its unique way. iNKT cells, a subset of innate-like T cells, serve as vital mediators bridging the innate and adaptive arms of the immune system. iNKT cells have features of both T cells and Natural Killer (NK) cells as they express an invariant TCR that can interact with the CD1d receptor, as well as NK1.1 and Ly49 receptors (56).

This unique feature allows iNKT cells to transcend the conventional boundaries between innate and adaptive responses due to their diverse surface marker expression. Furthermore, iNKT cells express a variety of receptors, including cytokine receptors such as IL-12R (122), as well as modulatory molecules like PD-1 and CD40l (123, 124). Additionally, they harbor cytotoxic molecules such as granzymes and perforins, providing them with a wide array of response capabilities tailored to specific contexts. iNKT cells play pivotal roles in modulating both innate and adaptive immune responses. They can activate or inhibit various cell populations on either side of the immune spectrum. Moreover, iNKT cells secrete distinct sets of cytokines and induce the proliferation of different subsets of cells, showcasing their remarkable versatility and significance in orchestrating the immune defense mechanisms.

## 6 iNKT cells and immune dysregulation in hematological cancers

Increased iNKT cells are found in various infectious diseases (1). Rarely, other pathological processes result in increased iNKT cells except in sickle cell crises (125, 126). Decreased iNKT cells correlate with the severity of several autoimmune diseases, cancer and GVHD (127–130). Of interest, decreased iNKT cells are associated with the poor outcome of bone marrow transplant (131), including the severity of GVHD although it is difficult to draw a conclusion whether the changes in number of iNKT cells are the cause or the consequence of these clinical settings (1).

From the perspective of tumor immunology, iNKT cells eliminate cancer by two main mechanisms (Figures 1B). The first mechanism is to directly recognize and eliminate cancer cells harboring CD1d via perforin/ granzyme, TNFα, TRAIL dependent apoptosis, FasL dependent apoptosis, or B cell mediated cytolysis (2, 39, 40, 66, 132–135). Particularly, CD1d is more commonly expressed in hematological malignancies as compared to non-hematological malignancies (39, 107, 136, 137). However, CD1d expression can be lost or diminished during disease progression (138, 139). This may have an unfavorable impact on tumor control. The other main mechanism that iNKT cells use to control tumor is indirectly crosstalk with other immune cells in the tumor microenvironment (TME) (2, 140). iNKT cells can promote dendritic cell maturation (1). iNKT cells can also secrete IL-12 which activates anti-tumor CD4+ and CD8+ T cells (1). Ultimately, iNKT cells can modulate myelomonocytic populations of tumor microenvironment to antagonize tumor progression (1, 2). Tumor associated macrophages (TAMs) can either promote anti-tumor activity (M1) or augment immune dysregulation (M2) leading to tumor progression or metastasis (99, 141). The dysregulation of TME can accelerate disease progression, render the survival advantages for cancer cells such as angiogenesis, invasion or proliferation, and interfere with or even negate therapeutic effect of cancer treatment including immunotherapy (2). The balance between tumor infiltrating T cells along with NK cells and immunosuppressing Treg shapes the outcome of immunologic interaction between immune cells and cancer cells (142). More importantly, 50% of tumor mass consists of tumor associated leukocytes such as tumor associated neutrophils (TANs), and tumor infiltrating myeloid-derived suppressor cells (MDSC) (143). iNKT cells can selectively eliminate CD1d expressing M2 like macrophages in a transgenic mouse model of CLL that delayed disease progression and improved survival (141, 144). On the contrary, tumors can evade immune response by upregulating the inhibitory NK receptor Ly49C/F/H/I resulting in unresponsive iNKT cells in murine prostate cancer model (145). The coadministration of IL-12 and α-GalCer can overcome this inhibitory signal (2). The upregulation of PD-1 on tumor unresponsive iNKT cells could also be thwarted by PD-1 or PD-L1 blockade. The coadministration of PD-1 or PD-L1 blockade along with iNKT agonist like Threitolceremide-6 is under investigation in the clinic for the treatment of patients with non-small cell lung carcinoma and melanoma (32). The PD1-CD28 CAR containing extracellular domain of PD1 fused to the intracellular co-stimulatory CD28 could also render the higher antitumor activity against PDLI+ lymphoma cells (146). Binding this CAR and its PDL1 transmits activating signals instead of inhibitory signals in lymphoma (146). Similar strategies addressing immune dysregulation associated with TME can also be potentially investigated in hematological malignancies to enhance anti-tumor activity.

iNKT cell therapy has been explored in the context of several autoimmune diseases. One aspect of potential therapeutic applications of iNKT based cell therapy is to utilize iNKT cells or engineered iNKT cells as either a preventive or treatment modality of graft-vs.-host disease (GVHD)—a life-threatening immunological complication that can occur after hematopoietic stem cell transplants (147). Donor derived T cells recognize and target recipient tissues, resulting in alloreactivity, tissue injury and destruction (147). Pro-inflammatory cytokines such as interferon-gamma (IFN-gamma) and tumor necrosis factor-alpha (TNF-alpha) aggravate GVHD whereas interleukin-4 (IL-4) and interleukin-10, which are anti-inflammatory cytokines suppress GVHD. Treg suppresses GVHD by producing interleukin-10 and transforming growth factor beta which can abrogate the activation and proliferation of T cells (148, 149). More importantly, there is growing evidence supporting the suppression or prevention of GVHD by iNKT cells through the expansion of Treg (150). The adoptive transfer of iNKT cells was 10–50x more potent than Treg in protecting mice from lethal GVHD in an identical model across major histocompatibility barriers (3). Further, the adoptive transfer of iNKT cells was effective in reducing pathology in a murine model of chronic GVHD (151). As such, iNKT based cell therapy can be potentially used to treat hematological malignancies and prevent or even treat GVHD simultaneously (152).

## 7 Potential clinical applications of iNKT cell therapy in hematological cancers

### 7.1 Dendritic cell vaccine (DC vaccine) and tumor-based vaccine

To overcome the limited therapeutic efficacy of administration of only α-GalCer due to anergy and unresponsive iNKT cells, the *ex vivo* stimulation and α-GalCer pulsed DC with or without tumor cells have been explored in several hematologic malignancies including multiple myeloma and lymphoma in murine models (152–156). This strategy improved survival and *in vivo* persistence of iNKT cells in a murine model. However, limited clinical benefits were observed in a clinical study (153). The administration of α-GalCer loaded tumor vaccines improved survival in murine models of B cell lymphoma, acute myeloid leukemia and multiple myeloma (153, 157–161). In addition, the tumor vaccine given after chemotherapy prevented the relapse of leukemia (161). Ultimately, clinical trials are required to endorse the clinical efficacy and validity of this approach.

### 7.2 Engineered iNKT cells utilizing chimeric antigen receptors (CAR)

**Figure 2:**
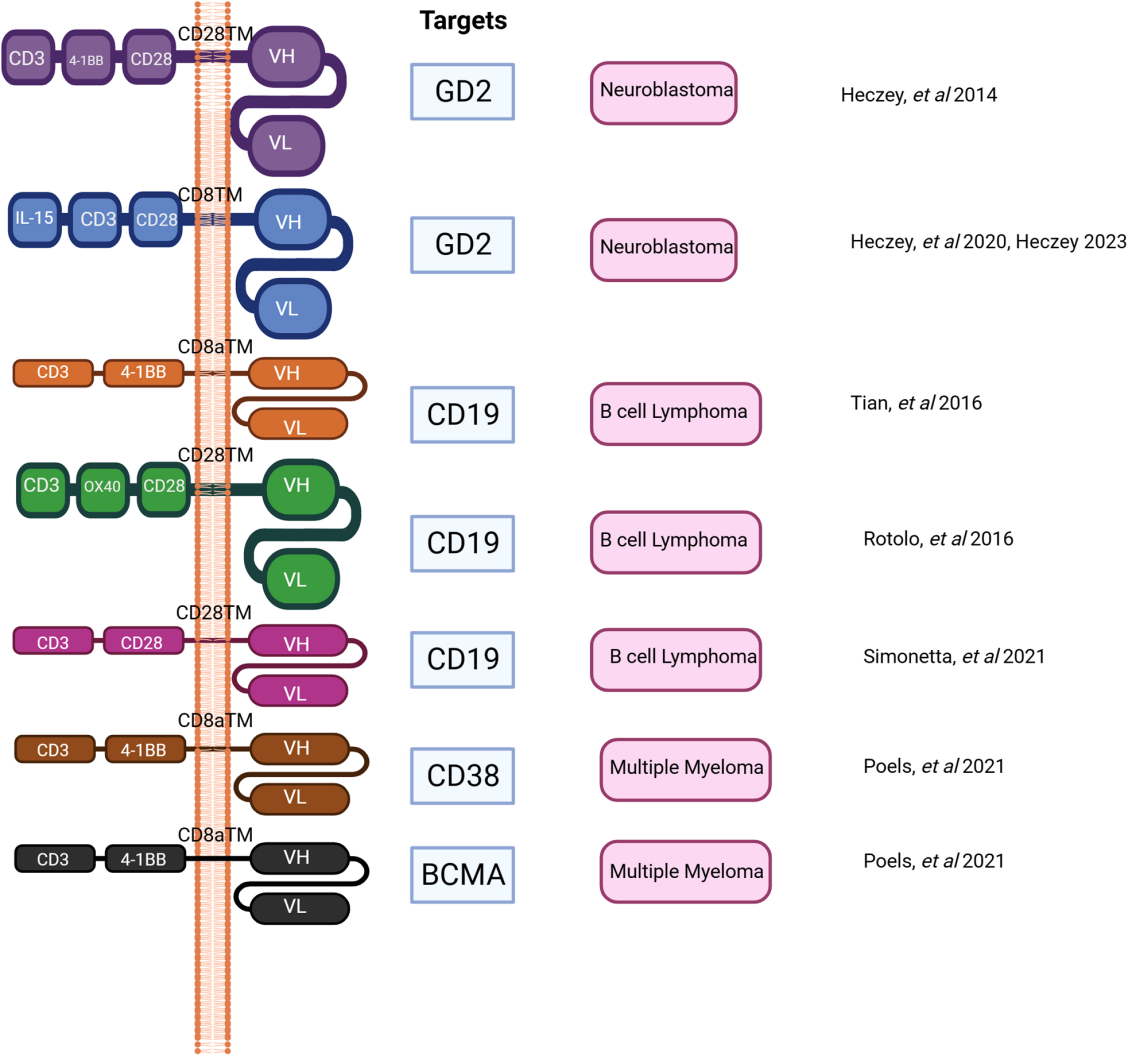
Shows the progress on CAR construct design in preclinical and clinical studies to date.

The utilization of adoptive transfer of *in vitro* expanded iNKT cells has been explored in both murine models and patients with hematological malignancies Tables 2, 3 (162–170) Figure 2 given the fact that it is feasible to expand iNKT cells *in vitro* and iNKT cells have inherent antitumor activity against CD1d cancer cells as well as immunosuppressing immune cells such as macrophages in TME (2, 4, 5, 140). Interestingly, Watarai and colleagues developed induced-pluripotent stem cells or iPSC—derived iNKT cells which retained the biological properties and their antitumor activity (171). Human CD34+ HSC derived iNKT (HSC-iNKT) cells also maintained their biological properties (172). In addition, these HSC-iNKT cells could protect against multiple myeloma and melanoma that expressed CD1d (172). Further investigation in clinical trials is required to confirm its clinical application which is poised to begin in 2024. iNKT cells have some unique beneficial biological properties which make them the ideal candidate for adoptive cell transfer approach to treat cancer: (1) they inhibit immunosuppressive myelomonocytic cells in TME via CD1d- cognate recognition (2), (2) the suppression of inhibitory signals from TME, and elimination of CD1d harboring cancer cells along with specific antitumor activity through CAR signaling likely to enhance therapeutic efficacy of CAR-iNKT cells through these synergistic mechanisms (2, 140). Rotolo and colleagues demonstrated that CAR iNKT cells against CD19 could exert better tumor control as compared to CAR T cells against CD19 through the synergistic interaction with CD1d and CD19 on lymphoma (173). In addition, CAR iNKT cells directed against CD19 could render the control of brain lymphomas (173). To maximize antitumor activity of iNKT cells, the adoptive transfer of CAR-iNKT has been investigated in several preclinical and phase I clinical studies (5, 7, 8, 173) (Figure 2).

**Table 2:** The advantages and disadvantages of utilization of various sources of cell-based therapies in hematological malignancies.

Cell type	Source	Advantage	Disadvantage	Immunologic interaction	Cell engraftment	Duration of response	Example
HSCs	Autologous/Allogenic	Graft-versus- leukemia or tumor (allogenic source)	Immunosuppression is required for allogenic source. Conditioning preparation is still required for autologous cell transplant. High dose chemotherapy is required (autologous and allogenic). Autologous HCT likely improves survival mainly in chemotherapy sensitive patients. GVHD (allogenic source)	Immunosuppression is required for allogenic source.	Potentially permanent	Long term, highly variable	HSCT for leukemia/lymphoma/multiple myeloma
T cell	Autologous/Allogenic	Known clinical efficacy against hematological malignancies (CAR-T cell)	Conditioning preparation is still required.	GVHD and potential rejection of treatment cells (for allogenic source, genetic modification is required)	Potentially permanent	Long term, highly variable	CAR-T both autologous source such as Tisagenlecleucel, Axicabtagene ciloleucel, Brexucabtagene autoleucel, and allogenic source such as UCART19, an allogeneic genome-edited anti-CD19 chimeric antigen receptor (CAR) T-cell in refractory B-cell ALL
			High cost of production.				
			The potential risk of GVHD (allogenic source, genetic modification is required). Potential CRS, neurotoxicity, and other immune mediated side effects.				
iNKT cell	Autologous/Allogenic	Do not cause GVHD.	A short *in vivo* persistence (This requires cytokine supplement or a vector design to augment its *in vivo* persistence).	Do not cause GVHD. iNKT cells may help to protect or even treat GVHD.	Potential transient.	Highly variable	CD19.IL15.CAR-iNKT for B cell malignancies. (clinical trial NCT04814004)
		Potential off-the-shelf product.					
		Known inherent anti-tumor activity against CD1d + tumors. iNKT cells can modulate myelomonocytic populations of tumor microenvironment to antagonize tumor progression.					
			A very small population of cells.				
			May require re-infusion to achieve a durable response,				
NK cell	Autologous/Allogenic	More manageable safety profiles as compared to T cell therapy. Potential off-the-shelf product.	Immunosupression in tumor microenvironment depresses NK cell anti-tumor activity and shortens its *in vivo* persistence.	Do not cause GVHD. It may suppress GVHD.	Potential transient.	Short term, highly variable	iC9/CD19-CAR-CD28-zeta2A-IL-15 NK cells for relapsed and refractory B cell malignancies (NCT03056339).
							FT500 (off-the-shelf iPSC NK cells) for advanced solid and hematological cancer (NCT03841110)

HSC, hematopoietic stem cells; HSCT, hematopoietic stem cell transplant; GVHD, graft versus host disease; iNKT cells, invariant natural killer T cells; NK, natural killer cells.

**Table 3:** Current clinical trials of iNKT/ engineered iNKT/CAR-iNKT for hematological malignancies and related diseases.

Clinical trial ID	Phase	Source	Indication	Route of administration	Intervention/Treatment/Product
NCT03774654	1	Allogenic	Relapsed or Refractory B-Cell Malignancies	IV	CD19.CAR-aNKT cells
NCT05487651	Phase 1 dose escalation	Allogenic	B-Cell Malignancies	IV	KUR-502 (CD19.CAR-aNKT cells) consists of transduced allogeneic natural killer T cells (aNKT) genetically modified with additional features to enhance their anti-tumor activity against CD19+ B-cell malignancies
NCT03605953	Not provided	Allogenic	GVHD	Not provided	Invariant NKT Cells for a Cell Immunotherapeutic Approach allowing the Control of Graft Versus Host-disease and preserving the Graft Versus Leukemia effect after Allogeneic Hematopoietic Stem Cell Transplantation
NCT04814004	1	Allogenic	Relapsed/Refractory/high-risk B-cell tumors	IV	hCD19.IL15.CAR-iNKT

Our group and others have demonstrated that the adoptive cell therapy approach utilizing CAR-iNKT cells could render survival benefits in murine models in hematological malignancies such as B cell lymphoma and multiple myeloma (10, 174). Simonetta and colleagues demonstrated that allogenic CAR iNKT could induce host CD8 T cell cross priming (10). As a result, this rendered durable antitumor activity beyond the physical presence of transferred CAR iNKT cells and this also improved tumor control in murine model (10).

**Figure 3:**
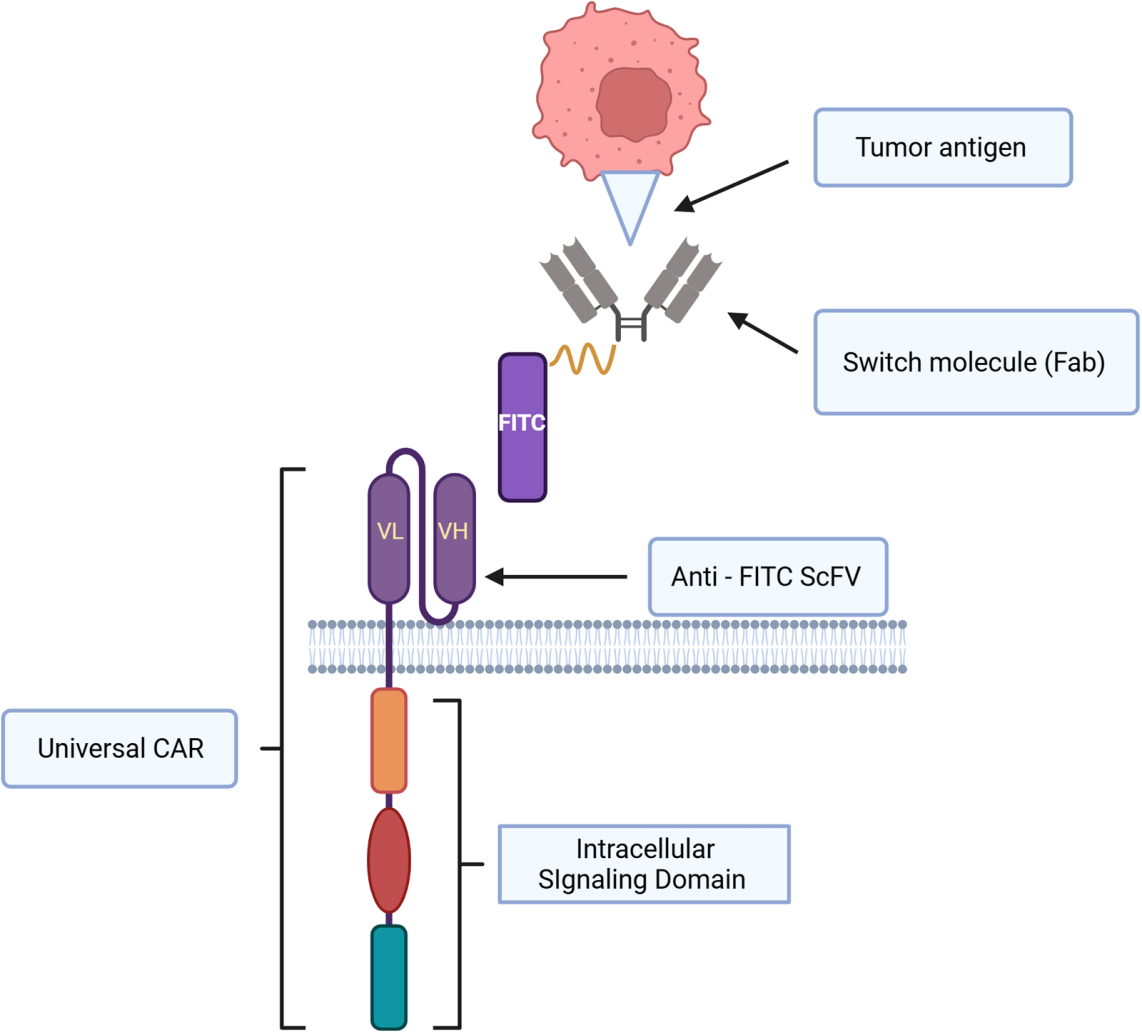
Schematic representation of universal CAR system design and immunological synapse of universal CAR, switch molecule and tumor antigen. This universal CAR-iNKT like CAR-T cell system consists of the flexible antigen binding component (e.g. svFC, monoclonal antibody or tumor specific ligand), and the iNKT cell signaling component.

CD62l+ CAR iNKT cells appeared to have prolonged *in vivo* survival and they were highly effective against B cell lymphoma, and neuroblastoma in NSG mice (5). Further, Ngai and colleagues found that the administration of IL-21 could protect and enhance CD62+ CAR iNKT's antitumor activity and increase survival in a murine model of lymphoma (6). CD4+ T cells and iNKT cells produce IL-21 (175, 176). IL-21 can abrogate apoptosis by increasing the expression of BCL-2. This improves survival of iNKT cells. IL21 also binds with motif YXXQ on IL-21R, then activates STAT3 pathway resulting in generating more memory cells and effector cells (6, 175, 177). Several studies demonstrated that CAR iNKT cells were effective against multiple myeloma and they prolonged survival in murine models. O'Neal and colleague showed that the co-administration of long-acting IL-7: rhIL-7-hyFc and CAR iNKT targeting BMCA improved anti-myeloma activity in a preclinical study (178). CAR iNKT cells were demonstrated to be safe and able to infiltrate tumors in patients with neuroblastoma in the recent update of a phase I clinical trial (8). In this clinical trial, autologous anti-GD2 CAR iNKT cell therapy against neuroblastoma exploiting the co-expression of GD2 and IL15 appeared to be safe with acceptable adverse events (8). Like fourth generation CAR T cells, CAR constructs can be designed to secrete additional cytokine to enhance antitumor activity, and in this clinical trial the co-expressing IL-15 promoted the development, expansion, and *in vivo* persistence of central memory iNKT cells (8, 163). The CAR-iNKT cell therapy in this clinical trial showed anti-tumor activity with two partial responses (2/12) and one complete response (1/12) in relapsed or refractory neuroblastoma (8). iNKT cells can also be engineered to acquire a second specific antigen by expressing recombinant TCRS that recognize tumor associated antigens, for example, human iNKT cells could be engineered with TCR specific for an HLA-2-restricted peptide epitope derived from melanoma such as MART or PRAME (2, 179). This iNKT-TCR demonstrated HLA-restricted antitumor activity in a xenogenic murine model (179). To further leverage the unique immunotherapeutic application of CAR-iNKT cells in hematological malignancies, a novel universal CAR platform can also be designed (Figure 3). In general, this universal CAR-iNKT cell system consists of the flexible antigen binding component (e.g., svFC, monoclonal antibody or tumor specific ligand), and the iNKT cell signaling component (180). This off-the-shelf, universal CAR platform is flexible, scalable, and amendable to diverse and multiple antigen specificity. The current FDA approved CAR T cells for hematological malignancies are autologous CAR T cells and CAR iNKT cells may be particularly attractive as an allogeneic product.

In the context of stem cell transplantation, iNKT cells mitigate the severity of GVHD and they maintain antitumor activity (151, 152). No evidence of GVHD was demonstrated in several murine models of hematological malignancies (152). Of interest, further modification to lower MHC expression on iNKT cells may be required to prevent host immunity from rejecting adoptive transferred allogenic iNKT cells, and this has been investigated in clinical trial of B cell lymphoma (2). Furthermore, the complete absence in MHC expression on allogenic gene edited iNKT cells may provoke “missing self” response by NK cell, and this will compromise the survival of allogenic gene edited iNKT cells (2). Hence, allogenic gene edited iNKT cells may require to be engineered to express ligands for the NK inhibitory receptors or they may require modification of CD47/SIRPα pathway to protect them from phagocytosis (2).

Like CAR T cell therapy—one of the historic successful trailblazers in cell therapy for hematological malignancies leading to the first FDA approved CAR T cell for precursor B cell acute lymphoblastic leukemia (pre-B ALL) (181) then followed by a number of other FDA approved treatments for pre-B ALL, B cell lymphoma, and multiple myeloma (182), CAR-iNKT cell based therapy can be adopted to enhance antitumor activity, improve survival, mitigate serious or decrease adverse reactions (2, 4, 8, 40, 180). We foresee that the advancement of vector design and refined treatment schedule to improve CAR iNKT *in vivo* persistence, and functionality resulting in better clinical outcomes. Further investigation to combine CAR iNKT based cell therapy with other treatment modalities to leverage its therapeutic efficacy may be warranted. For example, the combination of CAR iNKT cell-based therapy with traditional hematopoietic stem cell transplantation to achieve synergistic antitumor activity and minimize or prevent immunological complications such as GVHD (2, 152, 183). The combination of CAR iNKT cells with immune checkpoint blockade or small molecule such as ibrutinib to mitigate iNKT cell dysfunction and enhance anti-tumor activity like in CAR T cell therapy may increase therapeutic efficacy (163, 184, 185). The expected adverse effects such as cytokine release syndrome, neurotoxicity, or other unforeseen adverse effects in CAR -iNKT cells-based therapy and confirmation of their clinical application require additional further investigations in clinical trials.

### 7.3 iNKT cell therapy to prevent and treat GVHD

Graft-vs.-host disease (GVHD) is a major concern leading to high morbidity and mortality in patients undergone allogeneic hematopoietic cell transplantation (HCT) (186). The infused allografts during HCT contain mature CD4^+^ and CD8^+^ αβ T cells which establish hematopoietic engraftment, reconstitute T cells immunity, and induce graft-vs.-tumor (GVT) effect that is key for elimination of the malignant cells. However, these donor cells may recognize the host as “foreign” and invade the host tissues causing GVHD manifested as acute damages to the skin, gastrointestinal tract, and liver, termed acute GVHD, or as prolonged inflammation and immune dysregulation that could affect limited organs or be widespread, referred to as chronic GVHD (187–189). Immunosuppressive drugs are primarily employed as preventive or therapeutic measures to manage the complications presented by GVHD. But these are typically associated with severe toxic complications while do not always lead to complete resolution of GVHD manifestations (190–192). Therefore, a better understanding of the underlying mechanisms of immune dysregulation is necessary to overcome GVHD while maintaining appropriate GVT response, and thereby improve the outcome following HCT.

iNKT cells are one of the key immune regulatory cell populations extensively studied for their role in protection from GVHD. Murine studies of allogenic HCT by the group of Strober *et al*. demonstrated key role of host NKT cells in protection against GVHD following nonmyeloablative conditioning with total lymphoid irradiation (TLI) and anti-thymocyte serum (ATS) (193, 194). Our group employed adoptive transfer of CD4^+^ iNKT cells to major histocompatibility complex (MHC)-mismatched murine models of GVHD to demonstrate protective effects of donor-type (3, 195) as well as third-party (150) iNKT cells against GVHD. Furthermore, our group and others showed that specific activation of host iNKT cells using invariant T cell receptor (iTCR) stimulator a-galactosylceramide (a-GalCer) leads to mitigation of GVHD (30, 196, 197). Recently, we isolated highly purified murine iNKT sublineages: iNKT1, iNKT2 and iNKT17, and demonstrated antitumor function associated with iNKT1 while GVHD suppression function associated with iNKT2 and iNKT17 (42). These studies suggest iNKT cells enact key roles in the mechanisms underlying immune regulation leading to GVHD suppression. For the GVHD suppressive function of iNKT cells, their interplay with other immune regulatory cell populations is crucial. Multiple studies have provided compelling evidence that iNKT-induced interleukin-4 (IL4) dependent regulatory T (Treg) cell expansion drives inhibition of GVHD response in HCT (3, 30, 150, 198–201). Treg cells have potent immune suppressive function in allogeneic HCT with demonstrated capability to suppress GVHD while preserving the GVT effect (202–204). Further insights into the underlying mechanisms of tolerogenic immune pathways driving anti-GVHD response in HCT are provided by numerous murine studies implicating myeloid-derived suppressor cells (MDSCs) and CD8^+^ dendritic cells (DCs) in the interplay between Treg and iNKT cells (150, 205–207).

Various clinical studies validate iNKT cells being associated with GVHD suppressive function. Higher number of iNKT cells in the graft for allogeneic HCT is reported to be associated with lower risk of acute GVHD (128, 208, 209). Following allogeneic HCT, iNKT cells have widely been reported to persist and rapidly recover which is correlated with protection against GVHD. Post-HCT recovery analysis of iNKT cells demonstrated correlation between increased number of iNKT cells and protection against GVHD resulting in improved GVHD relapse free survival (129, 210). Likewise, in patients that received allogeneic HCT with total lymphoid irradiation (TLI) and anti-thymocyte globulin (ATG) conditioning, we revealed increased persistence of iNKT compared to CD4^+^ and CD8^+^ T cells post-TLI/ATG and protection against acute GVHD in patients with residual iNKT cells than those without detectable iNKT cells (211). To summarize, these studies suggest a key role of iNKT cells in suppression of GVHD in humans, and therefore attempts have been made to expand iNKT cells in HCT patients. A phase 2A study utilized RGI-2001, a liposomal formulation of a-GalCer to activate and expand iNKT cells which induced Treg expansion and reduced the incidence of grade 2 to 4 GVHD in the HCT patients (212). A recent study sheds light on a probable underlying mechanism of GVHD prevention by human iNKT cells where human culture-expanded iNKT cells were shown to induce DC apoptosis, and thereby impair the activation and proliferation of allo-reactive T cells (213). One of the key considerations in this regard could be the heterogeneity of iNKT cells, as evidenced by our own murine studies (42). Heterogeneity in human iNKT cells has been well characterized by a recent study where two iNKT phenotypes have been identified which correlated with a T helper 1 function and enhanced cytotoxic function (214). Identification of human iNKT subsets with Th2 function and ensuring their preservation during *in vitro* expansion could be crucial in clinical trials using iNKT cells. Despite a multitude of questions, the therapeutic potential of iNKT cells appear promising in overcoming a major limitation of HCT by achieving long-lasting suppression of GVHD.

## 8 Concluding remarks and future directions

The roles of iNKT cells in the specific immune mediated disease aspects are emerging, and the use of iNKT cells as a platform for cell therapy is beginning to be appreciated, with potential impact and roles in a wide range of diseases such as autoimmune diseases, infection, and especially cancer. CD1d restriction defines this cell population which is ubiquitously expressed and not polymorphic. In addition, CD1d is commonly expressed in hematological malignancies. Hence, iNKT cells are a potential candidate for “off-the-shelf” allogeneic cell therapy for hematological malignancies. iNKT cells have some unique beneficial biological properties which make them the ideal candidate for adoptive cell transfer to treat cancer such as the synergistic mechanism between the inherent anti-tumor activity against CD1d harboring cancer cells and the induction of host CD8+ T cell responses. The isolation, enrichment, and expansion of iNKT cells *in vitro* are feasible and iNKT cells can be readily transduced with viral approaches to express a CAR and as such are amendable as the “off-the-shelf” therapeutic agents for large-scale clinical application. We anticipate that the improved vector design and other strategies such as combinatorial treatment with small molecules or immune blockade inhibitor could improve CAR iNKT *in vivo* persistence, and its functionality, leverage its anti-tumor activity along with the abatement of iNKT cell dysfunction or exhaustion. These unique and beneficial biological properties along with several strategies adopted from development of CAR T cell therapy to address shortcomings make iNKT cell therapy an attractive novel therapeutic approach to address immune dysregulation and disease relapse in hematological malignancies.
